# Impact of Varied Dietary Macronutrient Ratios on the Nutrition Status of Swiss Albino Mice

**DOI:** 10.1002/fsn3.70957

**Published:** 2025-09-12

**Authors:** Hellen Kinyi, Charles Drago Kato, Gertrude N. Kiwanuka

**Affiliations:** ^1^ Department of Biochemistry, Faculty of Medicine Mbarara University of Science and Technology Mbarara Uganda; ^2^ Department of Biomedical Sciences, Medical College East Africa Aga Khan University Nairobi Kenya; ^3^ School of Biosecurity, Biotechnical and Laboratory Studies, College of Veterinary Medicine, Animal Resource and Biosecurity Makerere University Kampala Uganda

**Keywords:** dietary interventions, macronutrient composition, nutritional physiology, Swiss albino mice

## Abstract

The nutrition status of an organism depends on the balance between nutrient intake and utilization. Malnutrition results not only from inadequate caloric intake but also from imbalanced macronutrient ratios, which can affect aging, metabolism, and health. While many studies focus on individual nutrients, this study used purified, isocaloric diets with varying macronutrient ratios to assess dietary effects on nutrition markers in Swiss albino mice.

Mice were randomized into 6 dietary groups, each containing 6 males and 6 females housed separately. Each group received an isocaloric diet with different carbohydrate, protein, and lipid ratios for 15 weeks. Animal weight, body mass index (BMI), hematological indices, blood glucose, protein, and cholesterol levels were measured to assess the nutritional status.

Baseline mean body weight was 19.6 ± 0.43 g. Mice fed a high‐carbohydrate, low‐protein (HCLP) diet had the highest mean weight (32.83 ± 1.1 g) and BMI, whereas those on the high‐lipid, low‐protein (HLLP) diet had the lowest (25.9 ± 0.7 g). The high‐protein, low‐lipid (HPLL) diet had the highest RBC count (6.6 ± 0.1 × 10^12^ cells/L) and hemoglobin levels (17.5 ± 0.2 g/dL), while HLLP diets yielded the lowest fasting glucose (2.8 ± 0.3 mmol/L). Elevated serum cholesterol was observed in the HPLL group (180 ± 20 mg/dL).

Isocaloric purified diets with varied carbohydrate, protein, and lipid ratios differentially shaped growth, metabolic, and hematological outcomes in Swiss albino mice. High‐carbohydrate–low‐protein diet drove weight gain, protein restriction lowered red blood cell indices and serum proteins, low‐lipid diets raised cholesterol beyond normal, and high‐lipid–low‐protein diets reduced fasting glucose. We recommend that dietary macronutrient ratios should be tailored to specific physiological outcomes.

## Graphical abstract

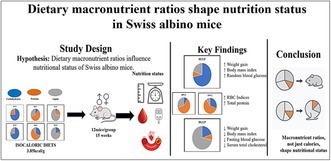

**Abbreviations**
AIN‐93M: American Institute of Nutrition‐1993 Maintenance diet
ALT: Alanine Transaminase
ANOVA: Analysis of Variance
BMI: Body Mass Index
CBC: Complete Blood Count
CHO: Carbohydrates
FBG: Fasting Blood Glucose
Hb: Hemoglobin
MCH: Mean corpuscular hemoglobin
MCV: Mean corpuscular volume
RBC: Red blood cells
RBG: Random Blood Glucose
SEM: Standard Error of Mean

## 1 Introduction

Nutrition status refers to an organism's overall health condition, shaped by a delicate balance between nutrient intake and utilization. This equilibrium enables the effective utilization of nutrients to sustain metabolic integrity, physiological functions, build reserves, and compensate for losses (Cacabelos 2016; Fernández‐Lázaro and Seco‐Calvo 2023). Depending on the adequacy of nutrient intake and utilization, particularly of macronutrients such as protein, carbohydrate, and lipid, an organism can be classified as well‐nourished or malnourished, with malnutrition encompassing both deficiencies and excesses (Espinosa‐Salas and Gonzalez‐Arias 2023).

According to the 2021 Global Nutrition Report, malnutrition from inadequate or excessive calorie intake is prevalent worldwide. Among children under 5 years old, 20% experience stunting, 6.7% suffer from wasting, and 5.7% are overweight. Globally, 2.2 billion adults grapple with overweight or obesity, 538.7 million have diabetes, and 1.2 billion contend with raised blood pressure (GNR 2021). The coronavirus disease (COVID‐19) pandemic further worsened the global nutrition crisis, as evidenced by increases in all forms of malnutrition (World Health Organization 2024). Many developing countries, including Uganda, are experiencing this double burden of malnutrition where both undernutrition and overnutrition coexist in the same households and communities (Ngaruiya et al. 2017). This is due to a shift to energy‐dense but nutrient‐poor diets that contribute to rising obesity and diet‐related noncommunicable diseases (Popkin 1998). These overlapping challenges highlight the urgent need to understand not only total caloric intake, but also how the relative proportions of macronutrients shape health outcomes.

Controlled animal models, such as laboratory mice, are particularly valuable for these investigations because they allow precise manipulation of diet composition while minimizing confounding factors present in human populations (Domínguez‐Oliva et al. 2023). Previous animal studies have shown that low‐protein diets impair growth and promote increased adiposity, whereas high‐protein diets tend to reduce fat mass and influence metabolic regulation (Chalvon‐Demersay et al. 2017). However, most of these studies have examined protein intake in isolation, often without considering the proportional contributions of carbohydrates and lipids under isocaloric conditions.

While the global prevalence of malnutrition highlights the critical impact of total calorie intake on health, research shows that malnutrition is not solely a consequence of overall caloric consumption (Howell and Kones 2017). The relative proportions of protein, carbohydrates, and lipids in the diet play a role in determining nutritional adequacy and influence key aspects of physiology, including aging, cellular metabolism, and disease risk (Cootes et al. 2022; Solon‐Biet et al. 2014; Venn 2020). This is because carbohydrates and lipids are the major sources of calories, while proteins have a significant influence on body composition, total energy intake, growth, reproduction, aging, susceptibility to metabolic disease, immune function, and resistance to diseases among others (Le Couteur et al. 2016; Nehme et al. 2023). Understanding how the macronutrient ratios affect physiological systems is therefore essential, and animal models provide a platform for studying these relationships under controlled dietary conditions.

Despite this, numerous studies investigating macronutrient impact on health often concentrate on individual nutrients, neglecting their interactive effects. Popular lifestyle diets such as Atkins and ketogenic regimens are often based on single‐nutrient perspectives, vilifying one macronutrient while promoting excessive consumption of the other two, and in doing so, disregarding the important roles all macronutrients play in organismal biochemistry (Ashtary‐Larky et al. 2021). Although some studies have explored the interactive effects of total calories, protein, and carbohydrates on lifespan and aging, they have overlooked the role of dietary lipids, which have several biological and dietary functions (Hew et al. 2016; Solon‐Biet et al. 2014; Solon‐Biet et al. 2015). Furthermore, many experiments employ complex cereal‐based diets whose heterogeneous composition makes it difficult to disentangle the influence of macronutrients from the effects of anti‐nutrients and other bioactive compounds.

To address these gaps, this study used purified diets containing varied proportions of protein, lipids, and carbohydrates to investigate their interactive effects on weight, body mass index (BMI), hematological markers, blood glucose, total protein, and total cholesterol in Swiss albino mice. By examining the physiological consequences of different macronutrient ratios in a controlled setting, this work aims to provide insights into dietary relationships that shape nutritional status and health outcomes.

## 2 Materials and Methods

### 2.1 Animals

A total of 20 male and female Swiss Albino mice were sourced from the Centre for Biosecurity and Global Health, College of Veterinary Medicine and Animal Resources, Makerere University. The animals were acclimatized to the laboratory environment for 2 weeks before random assignment into breeding groups, each comprising two females and one male. For the experimental phase, male and female Swiss Albino mice aged 6–8 weeks were randomly allocated to six dietary groups (Table 1), each containing twelve animals (six per sex).

**TABLE 1:** Macronutrient composition of experimental diets.

% Ratios	High CHO		High Protein		High Lipid	
	75C:20P:5 L	72C:8P:20 L	10C:60P:30 L	30C:60P:10 L	5C:20P:75 L	20C:8P:72 L
Experimental group	HCLL	HCLP	HPLC	HPLL	HLLC	HLLP
Ingredients	g	g	g	g	g	g
Corn starch	512	483	—	100	38.1	483
Casein	190.5	75.2	577.5	577.5	190.5	75.2
Maltodextrin	100	100	57.9	81	—	100
Sucrose	100	100	—	100	—	100
Soybean oil	21.4	85.5	140	42.8	320.9	85.5
Fiber	50	50	50	50	50	50
Mineral mix	35	35	35	35	35	35
Vitamin mix	10	10	10	10	10	10
L‐Cystine	1.8	1.8	1.8	1.8	1.8	1.8
Choline Bitartrate	2.5	2.5	2.5	2.5	2.5	2.5
Tert‐butylhydroquinone	0.008	0.008	0.008	0.008	0.008	0.008
Total	1023	953	875	1001	649	953
Total Calories (Kcal/g)	3.849	3.849	3.848	3.866	3.849	3.849

*Note:* Table 1 presents the formulation of the six experimental purified diets, each designed to emphasize a different macronutrient profile while maintaining comparable caloric density (3.85 kcal/g). Composition of AIN‐93 M adopted from (Reeves 1997).

Abbreviations: AIN, America Institute of Nutrition; CHO, Carbohydrates; g, grams; Kcal, HCLL, High Carbohydrate Low Lipid; HCLP, High Carbohydrate Low Protein; HPLC, High Protein Low Carbohydrate; HPLL, High Protein Low Lipid; HLLC, High Lipid Low Carbohydrate; HLLP, High Carbohydrate Low Protein.

### 2.2 Animal Housing and Welfare

Male and female mice were housed separately in metabolic cages (35 × 30 × 15 cm). Environmental conditions were controlled at 20°C–25°C with 70%–80% relative humidity with a 12‐h light/dark cycle. Beddings consisted of an inch of wood shavings, replaced weekly to minimize odor and pathogen growth. Fresh water and food were provided *ad libitum* daily.

Animals were observed once daily throughout the experiment for general health, activity, and behavior. Criteria predefined prior to the start of the study to minimize animal suffering included severe lethargy, inability to access food or water, rapid or labored breathing, and unresponsiveness to gentle stimulation. Body weight was measured biweekly throughout the study. In anticipation of potential adverse effects from extreme macronutrient imbalances, weight loss thresholds were predefined as a criterion for intervention: animals losing more than 20% of their initial body weight were closely monitored, and diet modification was considered if weight loss approached or exceeded 30%.

### 2.3 Ethics Statement

All procedures were conducted in accordance with the guidelines for the care and use of laboratory animals as outlined by (Garber et al. 2011), and were approved by the Mbarara University Institutional Ethics Committee (Study no. 19/08–20). The study was registered with the Uganda National Council of Science and Technology under registration number NS159ES.

### 2.4 Diets

Six isocaloric diets were formulated using food‐grade ingredients in accordance with the American Institute of Nutrition‐93 Maintenance Diet (AIN‐93 M) guidelines (Nielsen 2018; Reeves 1997). The vitamin and mineral mixes followed the standard AIN‐93 M composition, and their detailed contents are provided in Tables S1 and S2. In a pilot study, we described the local formulation of AIN‐93 M and demonstrated its safety and suitability as a standard diet for studies involving Swiss albino mice (Kinyi et al. 2023). For the experimental diets, we adopted macronutrient ratios associated with a maximum lifespan of 157 weeks and a ratio of 5% protein, 75% carbohydrates, and 20% lipids (Solon‐Biet et al. 2014).

These macronutrient ratios were used to formulate the experimental diets, each providing 3.8 kcal/g. However, within 4 weeks of diet initiation, both male and female mice fed diets containing 75% carbohydrates and 5% protein exhibited significant weight loss exceeding 30% of their initial body weight. This trend was not observed in the other experimental diet groups. Additionally, two animals in the HLLP group died. To mitigate these adverse effects, we modified the diets by increasing protein content to 8% in low‐protein diets and reducing it to 60% in high‐protein diets. The final composition of the experimental diets is presented in Table 1.

### 2.5 Assessment of Nutrition Status

Mice were fed experimental diets for 15 weeks. During this period, body weight was measured biweekly using a precision electronic balance (Kern PCB, Germany) and recorded to the nearest 0.1 g.

After 15 weeks, random blood glucose (RBG) was measured from tail blood using On‐Call Plus blood glucose test strips and a glucometer (ACON Laboratories, USA), as described by (Benedé‐Ubieto et al. 2020). For fasting blood glucose (FBG), animals were fasted for 6 h in the morning before measurement, using the same method.

Next, the animals were anesthetized via intraperitoneal injection using a mixture containing 16 mg of xylazine and 60 mg of ketamine as described by (Parasuraman et al. 2010). Adequate depth of anesthesia was confirmed by the absence of the pedal withdrawal reflex, lack of response to palpebral stimulation, and the presence of slow, regular respiration before any procedure was initiated.

While under deep anesthesia, body length (nasal‐to‐anal distance) was measured to the nearest 0.1 mm using a caliper, with animals placed in dorsal recumbency. The body mass index was calculated as the ratio between body weight and square surface area (g/m^2^) (Gargiulo et al. 2014).

Next, still under deep anesthesia, blood was collected by cardiac puncture into a purple‐top vacutainer containing ethylenediaminetetraacetic acid (EDTA) for hematology analysis and in a plain red‐top vacutainer for serum chemistry. Animals were euthanized by exsanguination under anesthesia, and death was confirmed by cessation of heartbeat and respiration.

Hematological parameters were determined using the Coulter CBC‐5 Hematology Analyzer (Beckman Coulter, China). Total protein and cholesterol levels were determined using the Beckman Coulter AU 480 Chemistry Analyzer following manufacturers' instructions provided with the respective kits, from Vitro Scient (Hannover, Germany).

### 2.6 Data Management and Analysis

Results from each experiment were recorded in an Excel spreadsheet and imported into Paleontological Statistics software (PAST), version 4.03, for analysis. Data for each group were first tested for normality using the Shapiro–Wilk test to determine whether parametric or nonparametric statistical methods were appropriate. Differences in group means were assessed using one‐way analysis of variance (ANOVA). When the assumptions of equal variances and homogeneity of variances were met, Tukey's post hoc test was applied; otherwise, Dunnett's multiple comparisons test was used. All results are expressed as mean ± standard error of the mean (SEM). Statistical significance was set at *p* < 0.05.

## 3 Results

### 3.1 Biweekly Animal Body Weight

**FIGURE 1:**
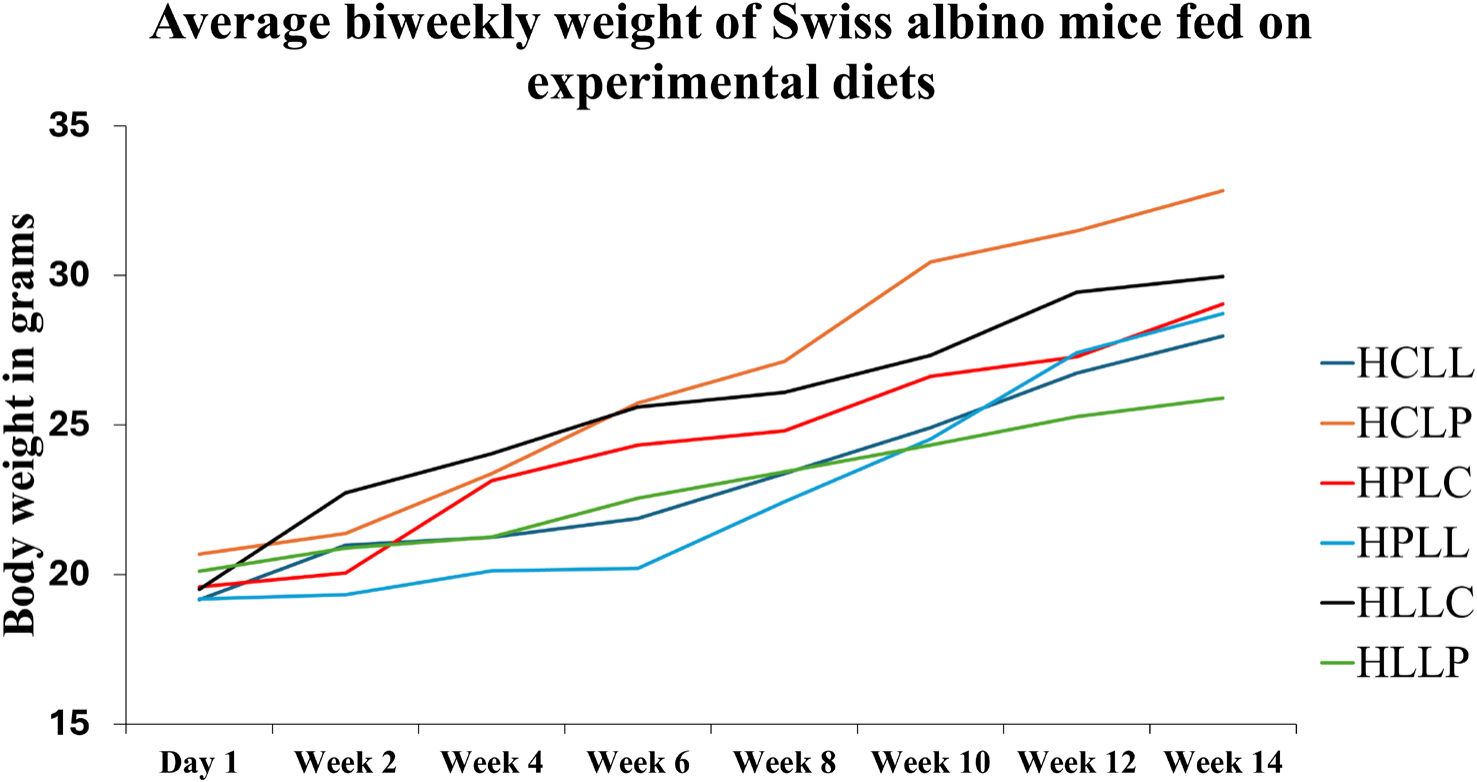
Average biweekly weight of Swiss albino mice fed experimental diets. HCLL—High carbohydrate low lipid, HCLP—High carbohydrate low protein, HPLC—High protein low carbohydrate, HPLL—High protein low lipid, HLLC—High lipid low carbohydrate, HLLP—High carbohydrate low protein.

Figure 1 shows the average biweekly weight of mice fed the experimental diets from day 1 to week 14. The average weight of the animals on day 1 was 19.6 ± 0.43 g, with no statistically significant differences among the diet groups (F_5, 66_ = 2.36, *p* = 0.47). Body weight increased progressively throughout the experimental period as seen in Figure 1. By week 14, statistically significant differences were observed among the six groups (F_5, 66_ = 7.92, *p* = 1.2 × 10^−5^). Mice fed the HCLP diet had the highest mean weight (32.83 ± 1.1 g), while those in the HLLP group had the lowest (25.9 ± 0.7 g). Significant differences emerged as early as week 2 in animals fed protein‐ and lipid‐rich diets (Table S3).

### 3.2 Body Mass Index of the Animals

**FIGURE 2:**
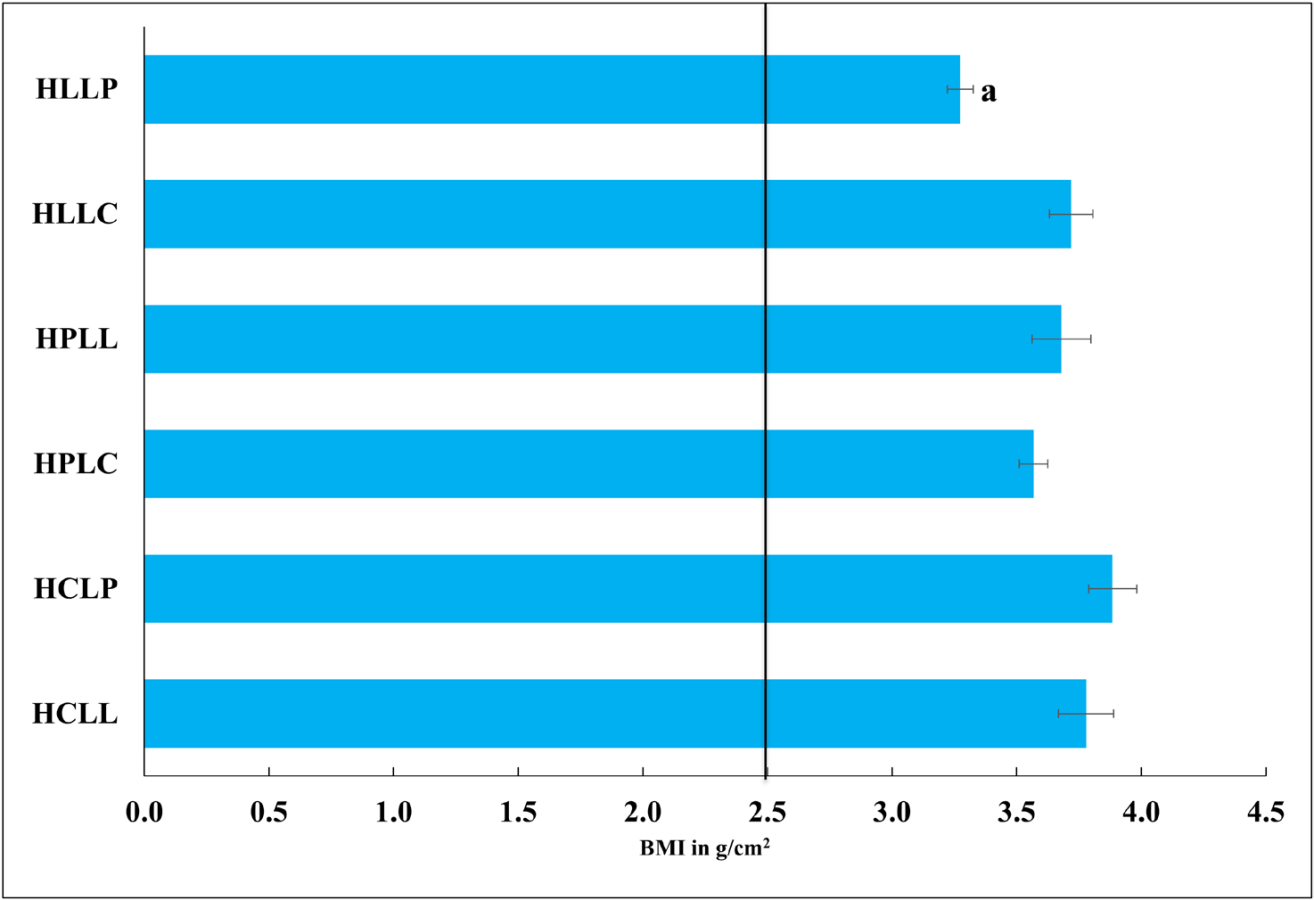
BMI of Swiss albino mice fed experimental diets for 15 weeks. The normal BMI range in mice is approximately 2.5–4.0 g/cm^3^. (a) Differs significantly from HCLL, HCLP, HPLL, and HLLC. HCLL—High Carbohydrate Low Lipid, HCLP—High Carbohydrate Low Protein, HPLC—High Protein Low Carbohydrate, HPLL—High Protein Low Lipid, HLLC—High Lipid Low Carbohydrate, HLLP—High Carbohydrate Low Protein.

Significant differences in body mass index (BMI) were observed among the diet groups on week 15 (F_5, 66_ = 3.07, *p* = 0.015). Animals fed the HCLP diet had the highest BMI (3.9 ± 0.1 g/cm^2^), while those in the HLLP group had the lowest BMI (3.3 ± 0.1 g/cm^2^) as shown in Figure 2. All the mice were within the normal reference range for mice of 2.5–4 g/cm^2^ (Kinyi et al. 2023).

### 3.3 Hematological Indices

There was a significant difference in mean RBC count among the groups at week 15 (F_5, 30_ = 17.1, *p* = 5.37 × 10^−8^). Animals fed low‐protein diets (HCLP and HLLP) had significantly lower mean RBC counts compared to those on high‐protein diets (Table 2). Similarly, mean corpuscular volume (MCV) differed significantly among the groups (F_5, 30_ = 5.01, *p* = 0.0019), with mice in the HCLP group showing lower MCV than those fed other diets. Mean hemoglobin concentration (Hb) also varied significantly across groups (F_5, 30_ = 10.13, *p* = 9.35 × 10^−6^). Animals in the HCLL and HCLP groups had significantly lower Hb levels compared to those on high‐protein and high lipid diets.

**TABLE 2:** Hematological indices of mice after 15 weeks on the six experimental diets.

Diet	HCLL	HCLP	HPLC	HPLL	HLLC	HLLP	*p*‐values
Mean RBC count 10^12^/L	6.3 ± 0.1	5.5 ± 0.1^a^	6 ± 0.1	6.6 ± 0.1	5.8 ± 0.1	5.1 ± 0.2^b^	5.37 × 10^−8^
Mean Corpuscular Volume (fL)	54.0 ± 1.0	48 ± 0.4^c^	53.3 ± 0.2	54.5 ± 1.8	52.9 ± 0.9	50.8 ± 1.5	0.0019
Mean Hematocrit (%)	32.1 ± 0.9	30 ± 0.7	31.9 ± 0.7	33.4 ± 0.6	30.7 ± 0.6	29.1 ± 0.6^d^	0.002
Mean Hemoglobin (g/dl)	15.5 ± 0.2^a^	14.7 ± 0.3^a^	17.4 ± 0.4	17.5 ± 0.2	16.4 ± 0.3	14.6 ± 0.8^a^	9.35 × 10^−6^
Mean Corpuscular Hemoglobin (pg)	24.8 ± 0.4^e^	23.6 ± 0.2^e^	29.1 ± 0.1	26.7 ± 0.4	28.5 ± 0.6	28.5 ± 0.4	5.45 × 10^−12^

Abbreviations: HCLL, High Carbohydrate Low Lipid; HCLP, High Carbohydrate Low Protein; HPLC, High Protein Low Carbohydrate; HPLL, High Protein Low Lipid; HLLC, High Lipid Low Carbohydrate; HLLP‐High Carbohydrate Low Protein.

^a^ significantly lower than HPLC and HPLL.

^b^ significantly lower than HPLC, HPLL, HCLL and HCLP.

^c^ significantly lower than HCLL, HPLC, HPLL, HLLC.

^d^ significantly lower than HPLC.

^e^ significantly lower than HPLC, HPLL, HLLC, HLLP.

### 3.4 Random and Fasting Blood Glucose

At week 15, random blood glucose levels differed significantly among the dietary groups (F_5,30_ = 5.44, *p* = 0.001). Mice fed the HCLP diet had the highest random blood glucose concentration (8.6 ± 0.5 mmol/L), which was significantly greater than that of the HCLL, HPLC, HPLL, and HLLP groups. Similarly, fasting blood glucose levels also varied significantly between groups (F _5,30_ = 5.98, *p* = 0.0006). The lowest values were recorded in the HLLP group (3.4 ± 0.3 mmol/L), which were significantly lower than those in the HCLP and HCLL groups, as shown in Table 3. Considering the normal physiological ranges for Swiss albino mice (random: 5–8 mmol/L; fasting: 4–7 mmol/L), the HCLP diet induced mild hyperglycemia under random feeding conditions, while the HLLP diet produced fasting hypoglycemia (Giknis et al. 2006).

**TABLE 3:** Effects of the six experimental diets on blood glucose, serum cholesterol, and serum cholesterol and proteins at 15 weeks.

Diet	HCLL	HCLP	HPLC	HPLL	HLLC	HLLP	*p*‐values
Random Blood Glucose (mmol/L)	6.5 ± 0.2	8.6 ± 0.5^a^	6.2 ± 0.2	6.2 ± 1.2	7.5 ± 1.5	5.9 ± 0.4	0.001
Fasting Blood Glucose (mmol/L)	5.9 ± 0.2	5.7 ± 0.3	4.9 ± 0.4	4.3 ± 0.7^b^	6.2 ± 0.5	3.4 ± 0.3^b^, ^c^	0.0006
Serum Total cholesterol (g/dl)	168.9 ± 31.3	127.7 ± 17.9	86.7 ± 14.3^e^	180 ± 20	120.8 ± 25.2	64.1 ± 3.8^d^	0.003
Total Protein (g/dl)	4.5 ± 0.1	4.5 ± 0.8	7.5 ± 0.7^f^	5.8 ± 0.5	6.0 ± 0.5	4.1 ± 0.2	0.001
Serum albumin (g/dl)	3.8 ± 0.1^g^	2.8 ± 0.3	3.5 ± 0.1	2.2 ± 0.3^h^	3.1 ± 0.1	2.9 ± 0.03	2.43 × 10^−5^

*Note:* Results presented as mean ± SEM.

Abbreviations: HCLL, High Carbohydrate Low Lipid; HCLP, High Carbohydrate Low Protein; HPLC, High Protein Low Carbohydrate; HPLL, High Protein Low Lipid; HLLC, High Lipid Low Carbohydrate; HLLP, High Carbohydrate Low Protein.

^a^ Significantly higher than HCLL, HPLC, HPLL, and HLLP.

^b^ Significantly lower than HLLC.

^c^ Significantly lower than HCLP and HCLL.

^d^ Significantly lower than HCLL and HPLL.

^e^ Significantly lower than HPLL.

^f^ Significantly higher than HCLL, HCLP, and HLLP.

^g^ Significantly higher than HCLP, HPLL, and HLLP from HPLL, HLLP, and HCLP.

^h^ Significantly lower than HPLL.

### 3.5 Total Proteins and Serum Albumin

Total protein and serum albumin levels also differed significantly across groups (F_5,30_ = 5.48, *p* = 0.001 and F_5,30_ = 9.23, *p* = 2.43 × 10^−5^, respectively). Mice in the HPLC group had the highest levels (7.5 ± 0.7 g/dL) of total protein, which was significantly greater than those in the HCLL, HCLP, and HLLP groups. Serum albumin was highest in the HCLL group (3.8 ± 0.1 g/dL) while the lowest was in the HPLL group (3.1 ± 0.1 g/dL) as shown in Table 3.

### 3.6 Total Cholesterol

Serum total cholesterol concentrations showed significant variation (F_5,30_ = 4.61, *p* = 0.003), with the lowest value in the HLLP group (64.1 ± 3.8 mg/dL), significantly lower than in the HCLL (168.9 ± 31.3 mg/dL) and HPLL (180.0 ± 20.0 mg/dL) groups. Female mice had higher cholesterol levels than males on the same diets (Figure S1, Supplementary file). The normal reference range for total serum cholesterol in healthy adult Swiss albino mice is approximately 80–120 mg/dL (Giknis et al. 2006), indicating that HCLL and HPLL diets elevated cholesterol above physiological levels, while HLLP levels were below the normal range.

### 3.7 Survival of Animals Fed on Experimental Diets

Mice fed diets HPLC, HPLL, HLLC, and HCLP exhibited a 100% survival through the 15 weeks of the study. Animals in the HCLL had a survival rate of 92%. The lowest survival rate of 89% was exhibited by animals in group HLLP. Despite these variations, statistical analysis using Kaplan–Meier analysis did not reveal significant differences between the groups. The log‐rank *p*‐value was calculated as 0.05 (p (χ^2^) = 0.95), indicating that the observed differences in survival rates were not statistically significant.

## 4 Discussion

Recognizing that the constituents of one's diet play a pivotal role in shaping health, this study analyzed the interactive effects of dietary macronutrients on health outcomes of Swiss albino mice maintained on purified diets for 15 weeks. The factors influencing nutritional choices and intake are diverse, encompassing genetic variations, taste preferences, satiety, hormones, and pathological changes (Kelly et al. 2019; Solon‐Biet et al. 2014). To control for these variables, we selected healthy Swiss albino mice aged 6–8 weeks and employed isocaloric diets based on AIN‐93 M nutrients to assess the influence of macronutrient ratios.

Initial findings indicated that both high and low dietary protein exert significant but contrasting effects on the nutritional wellbeing of Swiss Albino mice. High‐protein intake promotes weight loss and reduced appetite, effects that may be advantageous in the management of obesity but detrimental in physiological states that require sustained growth or adequate energy balance (Cuenca‐Sánchez et al. 2015). These outcomes are attributed to enhanced satiety associated with high blood amino acid concentrations, elevated blood ketone levels, and increased energy expenditure triggered by diet‐induced thermogenesis (Bensaïd et al. 2002; Khan et al. 2021). Furthermore, high‐protein diets stimulate the production of anorexigenic hormones such as glucagon‐like peptide (GLP‐1), cholecystokinin (CCK), and peptide tyrosine‐tyrosine (PYY), while suppressing the production of the hunger‐stimulating hormone Ghrelin (Batterham et al. 2002; Khan et al. 2021; Moon and Koh 2020). Conversely, very low protein diets (1%–5%) have also been associated with low body weight and appetite suppression (Masuoka et al. 2020; Wu et al. 2021). This effect may be explained by the depletion of adipose tissue and reduced leptin secretion, leading to insufficient stimulation of hypothalamic hunger pathways (Wu et al. 2021). Together, these findings highlight that deviations in either direction, excessively high or low dietary protein, can compromise nutritional status.

The ‘calories in and calories’ out paradigm simplistically defines an imbalance of calories as the reason for body weight fluctuations (Howell and Kones 2017; Minderis et al. 2021). However, despite all experimental diets being isocaloric, progressive differences in body weight and BMI suggest that macronutrient composition influenced growth. Diets containing 3%–8% protein such as HCLP have been shown to activate orexigenic pathways through the hypothalamic regulation of appetite, a phenomenon explained by the protein leverage hypothesis (Tomé et al. 2020). This adaptive increase in food intake compensates for protein dilution in the diet, leading to higher total energy consumption despite identical caloric density (Raubenheimer and Simpson 2023). In addition, high dietary carbohydrate availability promotes efficient lipogenesis and glycogen storage, further supporting weight gain (Le Couteur et al. 2016).

In contrast, the low body weight and BMI recorded in the HLLP group align with findings from previous studies in which mice were fed lipid‐rich, protein‐deficient diets (Rang et al. 2021; Wu et al. 2021). This outcome may be explained by the carbohydrate–insulin hypothesis whereby carbohydrate restriction reduces postprandial insulin secretion, enhancing lipolysis and fatty acid oxidation while limiting lipid storage (Friedman and Appel 2019). Furthermore, ketogenic‐like diets have been associated with increased thermogenesis and energy expenditure, possibly through brown adipose tissue activation and mitochondrial uncoupling, which may further contribute to low weight and BMI (Srivastava et al. 2013).

Intermediate weight and BMI outcomes in the other diet groups suggest a balance between the anabolic effects of adequate protein and carbohydrate supply and the increased energy expenditure or satiety effects of higher fat content. Collectively, these findings highlight that, even in isocaloric conditions, macronutrient ratios can drive divergent weight trajectories through their effects on appetite regulation, substrate utilization, and thermogenic pathways.

Although nutritional anemia is most frequently attributed to deficiencies of iron, folate, and cobalamin, macronutrient composition can also substantially influence hematological status (Tateishi et al. 2023). Our results indicate that Swiss albino mice fed low dietary protein (8%) for 15 weeks have lower hematological indices. Similar findings were found in rats fed a diet containing 4.5% protein for 60 days (Lewicki et al. 2014). This reduction may be explained by the role of dietary protein as a source of essential amino acids required for the synthesis of erythropoietin as well as hemoglobin and membrane proteins of red blood cells (Church et al. 2020). Inadequate protein intake can therefore impair erythropoiesis and reduce overall hematological indices.

Low hemoglobin level is also a common feature of protein deficiency in humans (Bianchi 2016; Keller 2019). Protein deficiency reduces the availability of essential amino acids required for globin synthesis, thereby impairing hemoglobin assembly even when iron supply is adequate (Rahim et al. 2023). Additionally, chronic low protein intake may suppress hepatic production of transferrin and other iron transport proteins, further limiting erythropoiesis (Moustarah and Daley 2024). Reduced MCV observed in HCLP mice suggests microcytosis, which can arise from impaired hemoglobin synthesis or defective erythrocyte maturation (Lolascon et al. 2009). Interestingly, lipid and carbohydrate‐rich diets with low protein content not only lowered Hb and RBC counts but also reduced mean corpuscular hemoglobin (MCH). This suggests that macronutrient imbalance can influence both the number and oxygen‐carrying capacity of erythrocytes. HCLP appeared particularly detrimental, possibly due to the combined effects of amino acid scarcity and the metabolic stress of high glycemic load, which may increase oxidative damage to red cells (Tan et al. 2018). These results highlight that adequate dietary protein is essential for maintaining healthy hematological indices, and that diets skewed toward carbohydrates or lipids at the expense of protein could increase the risk of anemia and impaired oxygen transport capacity, even in the absence of classic micronutrient deficiencies.

Blood glucose measurements, such as random blood sugar (RBG), allow the identification of fluctuations in circulating glucose, primarily derived from dietary intake, in response to diet, physical activity, and disease processes (Bowen et al. 2015). In this study, protein content appeared to be a primary driver as animals on the HCLP diet had the highest RBG. This aligns with the compensatory feeding hypothesis, whereby protein dilution increases food intake, disproportionately raising carbohydrate consumption and postprandial glycaemia (Le Couteur, Solon‐Biet, Cogger, et al. 2016). Chronic exposure to such elevated RBG may predispose individuals to insulin resistance and altered lipid metabolism over time (Biobaku et al. 2019).

In contrast, the HLLP group had the lowest FBG, likely due to insufficient dietary amino acids to sustain gluconeogenesis during fasting, resulting in reduced basal glucose availability (Moon and Koh 2020; Pesta and Samuel 2014). This pattern reflects the critical role of protein in maintaining glucose homeostasis during fasting periods, and suggests that extreme macronutrient imbalances can impair both fed and fasted state glycemic control.

The need to manage blood cholesterol levels is critical, given their association with multiple noncommunicable diseases (Coelho‐Júnior et al. 2023). Diets rich in animal proteins, such as casein, have been linked to elevated cholesterol in both animals and humans (Koury et al. 2014; Wang et al. 2022). In this study, the sole lipid source was soybean oil, which is high in linoleic and linolenic acids, which are polyunsaturated fatty acids with established hypocholesterolemic effects (Sasidharan et al. 2013). Although the precise mechanisms remain unclear, polyunsaturated fatty acids are thought to reduce hepatic cholesterol synthesis by modulating Sterol Regulatory Element‐binding Protein (SREBP)‐1 and SREBP‐2 activity and enhancing low‐density lipoprotein receptor expression, leading to greater clearance of circulating cholesterol (Schwab et al. 2014). Our findings reflect this: the lowest cholesterol levels occurred in mice fed HLLC and HLLP diets, both high in lipid content, suggesting that the higher proportion of polyunsaturated fatty acids outweighed the cholesterol‐raising effects of casein protein. Conversely, cholesterol was highest in the HPLC diet, where lipid content was relatively low but protein was high, consistent with evidence that high dietary protein from casein can elevate serum cholesterol. Notably, female mice in this study showed higher serum cholesterol levels compared to males fed similar diets, which could be due to the increased expression of cholesterologenic genes in female mice fed low‐cholesterol diets (Lorbek et al. 2013).

Despite varied responses in the different physiological parameters assessed, there was no significant difference in the survival of animals fed the experimental diets for 15 weeks. This could be due to a lack of variation in the total dietary calories, which has been reported to influence lifespan and health (Fontana and Partridge 2015; Mattison et al. 2017). Lifespan and long‐term health outcomes are complex and multifactorial, and their manifestations may require extended periods to become evident. While our study design allowed us to assess various physiological parameters, the full impact of dietary composition on survival may necessitate more extended observation.

## 5 Conclusions and Recommendations

This study demonstrates that the balance of dietary macronutrients strongly influences growth, metabolic, and hematological outcomes in Swiss albino mice. Diets high in carbohydrates but low in protein promoted weight gain and a higher body mass index, while adequate protein was essential for supporting healthy blood cell counts and proteins. Lipid‐rich diets containing soybean oil lowered serum cholesterol, consistent with the hypocholesterolemic effects of polyunsaturated fatty acids, whereas low‐lipid diets elevated cholesterol beyond physiological limits. High‐lipid–low‐protein diets also reduced fasting glucose. Sex‐related differences were evident, with females exhibiting higher cholesterol than males on similar diets, suggesting hormonal or genetic influences.

These findings highlight potential risks in populations consuming predominantly high‐carbohydrate, low‐protein diets, including susceptibility to weight gain, anemia, and impaired glycemic control. Although short‐term survival was unaffected, the physiological shifts observed indicate the significance of macronutrient ratios. Importantly, no single macronutrient ratio optimally supported all traits. Instead, different nutrient profiles selectively enhanced growth, blood indices, lipid regulation, or glucose control. We therefore recommend that dietary macronutrient ratios be tailored to the specific physiological outcomes desired, whether in experimental design, clinical nutrition, or public health interventions.

## 6 Strengths and Limitations of the Study

A key strength of this study is its controlled experimental design, which used purified diets with precisely defined macronutrient compositions, allowing clear attribution of observed effects to specific dietary components. The use of Swiss albino mice, a well‐characterized model for nutritional and metabolic research, enhances reproducibility and comparability with other studies. In addition, the study evaluated multiple biochemical and physiological markers, providing a comprehensive assessment of the dietary effects beyond single endpoints.

This study was conducted over a 15‐week period, a relatively short time frame that may not capture long‐term effects of dietary interventions, especially in relation to lifespan. Nevertheless, the findings justify longer term studies for a better understanding of the impact of these diets on other physiological statuses. Diets in this study contained specific sources of macronutrients (e.g., casein for protein, soybean oil for lipids). Real‐world diets are much more complex and varied, which can make it challenging to draw direct parallels between the study diets and typical human diets.

## Author Contributions

**Charles Drago Kato:** conceptualization (equal), methodology (equal), supervision (supporting), validation (supporting), writing – review and editing (supporting). **Gertrude N. Kiwanuka:** conceptualization (equal), methodology (equal), project administration (equal), supervision (lead), writing – review and editing (supporting). Hellen Kinyi: Conceptualization (equal), methodology (equal), Investigation, Data curation, formal analysis, writing original draft, reviewing and editing.

## Ethics Statement

Protocols and procedures were approved by the Mbarara University Institutional Ethics Committee (Study no. 19/08–20). The study was registered with the Uganda National Council of Science and Technology under registration number NS159ES.

## Conflicts of Interest

The authors declare no conflicts of interest.

## Supporting information


**Table S1:** Composition of the AIN‐93 M mineral mix used in experimental diets.
**Table S2:** Composition of the AIN‐93 M vitamin mix used in experimental diets.
**Table S3:** Mean biweekly weights (g) of mice fed experimental diets
**Figure S1:** Total Cholesterol of Swiss albino mice fed experimental diets for 15 weeks
**Table S4:** P‐values of Tukey's pairwise comparison of total cholesterol of male and female mice fed experimental diets for 15 weeks

## Data Availability

The datasets used and/or analysed during the current study are included in the manuscript.
