# Supplementary material for: Impact of Varied Dietary Macronutrient Ratios on the Nutrition Status of Swiss Albino Mice
Source: Food Sci Nutr. 2025 Sep 12;13(9):e70957. doi: 10.1002/fsn3.70957 (PMC12426907; doi:10.1002/fsn3.70957)
Supplement: Supplementary file 1 — Table S1: Composition of the AIN‐93 M mineral mix used in experimental diets. Table S2: Composition of the AIN‐93 M vitamin mix used in experimental diets. Table S3: Mean biweekly weights (g) of mice fed experimental diets Figure S1: Total Cholesterol of Swiss albino mice fed experimental diets for 15 weeks Table S4: P‐values of Tukey's pairwise comparison of total cholesterol of male and female mice fed experimental diets for 15 weeks [file FSN3-13-e70957-s001.docx]

**Supplemental Information**

**Impact of varied dietary macronutrient ratios on the nutrition status of Swiss albino mice**

Authors: Hellen Kinyi, Charles Drago Kato and Gertrude N. Kiwanuka

**INDEX TO SUPPLEMENTAL INFORMATION**

Table S1: Composition of the AIN-93M mineral mix used in experimental diets.

Table S2: Composition of the AIN-93M vitamin mix used in experimental diets.

Table S3: Mean biweekly weights (g) of mice fed on experimental diets

Figure S1: Total Cholesterol of Swiss albino mice fed on experimental diets for 15 weeks

Table S4: P-values of Tukey's pairwise comparison of total cholesterol of male and female mice fed on experimental diets for 15 weeks

**Table S1 related to Table 1:****Composition of the AIN-93M mineral mix used in experimental diets.**

| Ingredient | Amount (g/kg mix) |
| --- | --- |
| Calcium carbonate | 357.0 |
| Potassium phosphate, monobasic | 250.0 |
| Potassium citrate, monohydrate | 28.00 |
| Sodium chloride | 74.0 |
| Potassium sulphate | 46.6 |
| Magnesium oxide | 24.0 |
| Ferric citrate | 6.06 |
| Zinc carbonate | 1.65 |
| Manganous carbonate | 0.63 |
| Cupric carbonate | 0.30 |
| Potassium iodate | 0.01 |
| Sodium selenate | 0.01025 |
| Ammonium paramolybdate | 0.00795 |
| Sodium metasilicate | 1.45 |
| Chromium potassium sulphate | 0.275 |
| Lithium chloride | 0.0174 |
| Boric acid | 0.0815 |
| Sodium fluoride | 0.0635 |
| Nickel carbonate | 0.0318 |
| Ammonium vanadate | 0.0066 |
| Powdered sucrose | 209.806 |

Values were taken from (Reeves 1997)

**Table S2 related to Table 1:****Composition of the AIN-93M vitamin mix used in experimental diets.**

| Ingredient | Amount (g/kg mix) |
| --- | --- |
| Nicotinic acid | 3.0 |
| Calcium pantothenate | 1.6 |
| Pyridoxine HCl | 0.7 |
| Thiamine HCl | 0.6 |
| Riboflavin | 0.6 |
| Folic acid | 0.2 |
| Biotin | 0.02 |
| Vitamin B₁₂ | 2.5 |
| Vitamin E | 15.0 |
| Vitamin A palmitate | 0.8 |
| Vitamin D₃ | 0.25 |
| Vitamin K₁ | 0.075 |
| Powdered Sucrose | 974.655 |

**Table S3 related to Figure 1**: Mean biweekly weights (g) of mice fed on experimental diets

|  | **HCLL** | **HCLP** | **HPLC** | **HPLL** | **HLLC** | **HLLP** |
| --- | --- | --- | --- | --- | --- | --- |
| **% ratios** | **75C:20P:5L** | **72C:8P:20L** | **10C:60P:30L** | **30C:60P:10L** | **5C:20P:75L** | **20C:8P:72L** |
| Day 1 | 19.16 ± 0.6 | 20.68 ± 0.4 | 19.59 ± 0.5 | 19.18 ± 0.7 | 19.5 ± 0.5 | 20.1 ± 0.8 |
| Week 2 | 20.98 ± 0.5 | 21.38 ± 0.7 | 20.05 ± 0.3* | 19.33 ± 0.3 | 22.73 ± 0.7* | 20.89 ± 0.3 |
| Week 4 | 21.24 ± 1.9 | 23.38 ± 0.5 | 23.14 ± 0.5* | 20.13 ± 1.0 | 24.04 ± 0.7* | 21.25 ± 1.2 |
| Week 6 | 21.88 ± 0.6 | 25.73 ± 0.6* | 24.33 ± 0.6* | 20.21 ± 0.4 | 25.6 ± 0.8* | 22.55 ± 0.9 |
| Week 8 | 23.38 ± 0.9 | 27.13 ± 0.7* | 24.81 ± 0.6* | 22.44 ± 0.6 | 26.09 ± 1.1* | 23.43 ± 1.2 |
| Week 10 | 24.91 ± 0.9* | 30.45 ± 1.1* | 26.63 ± 0.9* | 24.53 ± 0.9* | 27.33 ± 0.9* | 24.33 ± 1.1* |
| Week 12 | 26.73 ± 1.3* | 31.49 ± 0.9* | 27.28 ± 0.8* | 27.41 ± 0.9* | 29.44 ± 1.1* | 25.28 ± 0.9* |
| Week 14 | 27.98 ± 1.2* | 32.83 ± 1.1* | 29.05 ± 0.7* | 28.72 ± 1.1* | 29.97 ± 1.0* | 25.9 ± 0.7* |

Results are presented as mean ± SEM. Asterix indicate significant differences from day 1 in same group. (Tukey’s pairwise multiple comparisons, P < 0.05).

**Key:** **HCLL**-High Carbohydrate Low Lipid, **HCLP**-High Carbohydrate Low Protein, **HPLC**-High Protein Low Carbohydrate, **HPLL**-High Protein Low Lipid, **HLLC**-High Lipid Low Carbohydrate, **HLLP**-High Carbohydrate Low Protein

**Figure S1: Total Cholesterol of Swiss albino mice fed on experimental diets for 15 weeks**

Graphs are presented as mean ± SEM. Asterix indicate significant differences from males in same group. (Tukey’s pairwise multiple comparisons, P < 0.05).

**Key:** **HCLL**-High Carbohydrate Low Lipid, **HCLP**-High Carbohydrate Low Protein, **HPLC**-High Protein Low Carbohydrate, **HPLL**-High Protein Low Lipid, **HLLC**-High Lipid Low Carbohydrate, **HLLP**-High Carbohydrate Low Protein

**Table S4 related to Table 3:** P-values of Tukey's pairwise comparison total cholesterol of male and female mice fed on experimental diets for 15 weeks p ˂ 0.05.

|  |  | **HCLL** | | **HCLP** | | **HPLC** | | **HPLL** | | **HLLC** | | **HLLP** | |
| --- | --- | --- | --- | --- | --- | --- | --- | --- | --- | --- | --- | --- | --- |
|  |  | Male | Female | Male | Female | Male | Female | Male | Female | Male | Female | Male | Female |
| **HCLL** | Male |  | 0.005893 | 1 | 0.8458 | 0.9998 | 0.9998 | 0.7133 | 0.07978 | 0.9493 | 0.4146 | 0.7777 | 0.992 |
|  | Female | 6.467 |  | 0.004025 | 0.2187 | 0.001049 | 0.001049 | 0.3274 | 0.9999 | 0.000215 | 0.6131 | 7.85E-05 | 0.000452 |
| **HCLP** | Male | 0.2309 | 6.698 |  | 0.7625 | 1 | 1 | 0.6132 | 0.05902 | 0.9781 | 0.3275 | 0.8582 | 0.9979 |
|  | Female | 2.42 | 4.047 | 2.651 |  | 0.4167 | 0.4167 | 1 | 0.7673 | 0.1365 | 0.9997 | 0.05712 | 0.2412 |
| **HPLC** | Male | 1.039 | 7.506 | 0.8084 | 3.459 |  | 1 | 0.2884 | 0.01939 | 0.9999 | 0.1213 | 0.9909 | 1 |
|  | Female | 1.039 | 7.506 | 0.8084 | 3.459 | 0 |  | 0.2884 | 0.01939 | 0.9999 | 0.1213 | 0.9909 | 1 |
| **HPLL** | Male | 2.772 | 3.695 | 3.002 | 0.3516 | 3.811 | 3.811 |  | 0.8752 | 0.0839 | 1 | 0.03352 | 0.1556 |
|  | Female | 4.803 | 0.9813 | 5.009 | 2.638 | 5.732 | 5.732 | 2.324 |  | 0.004825 | 0.9831 | 0.001935 | 0.009348 |
| **HLLC** | Male | 1.996 | 8.463 | 1.765 | 4.416 | 0.9569 | 0.9569 | 4.768 | 6.588 |  | 0.02974 | 1 | 1 |
|  | Female | 3.464 | 3.003 | 3.695 | 1.044 | 4.504 | 4.504 | 0.6927 | 1.704 | 5.46 |  | 0.01117 | 0.05899 |
| **HLLP** | Male | 2.612 | 9.079 | 2.381 | 5.032 | 1.572 | 1.572 | 5.383 | 7.139 | 0.6154 | 6.076 |  | 0.9997 |
|  | Female | 1.546 | 8.013 | 1.315 | 3.966 | 0.5063 | 0.5063 | 4.317 | 6.185 | 0.4505 | 5.01 | 1.066 |  |

**Key:** **HCLL**-High Carbohydrate Low Lipid, **HCLP**-High Carbohydrate Low Protein, **HPLC**-High Protein Low Carbohydrate, **HPLL**-High Protein Low Lipid, **HLLC**-High Lipid Low Carbohydrate, **HLLP**-High Carbohydrate Low Protein
